# PEEP levels in COVID-19 pneumonia

**DOI:** 10.1186/s13054-020-03049-4

**Published:** 2020-06-06

**Authors:** Vasiliki Tsolaki, Ilias Siempos, Eleni Magira, Stelios Kokkoris, George E. Zakynthinos, Spyros Zakynthinos

**Affiliations:** 1 Intensive Care Unit, University Hospital of Larissa, Faculty of Medicine, University of Thessaly, Larissa, Greece; 2 Intensive Care Unit, Evangelismos General Hospital, National and Kapodistrian University of Athens Medical School, Athens, Greece

To the Editor:

Recently, the Surviving Sepsis Campaign COVID-19 guidelines and ATS suggest that a ventilatory strategy complying with the ARDSnet protocol should be applied to manage COVID-19 pneumonia [1–3]. However, “COVID-19 lung” pathophysiology seems to be divergent from the “ARDS lung”; hence, heart-lung interactions may be more pronounced than initially considered [2, 3].

We studied 17 patients (March 20–April 14, 2020) treated in two Greek University Intensive Care Units. Patients had COVID-19 pneumonia fulfilling the Berlin criteria of acute respiratory distress syndrome (ARDS) and were on the 2nd or 3rd day of invasive mechanical ventilation. Positive end-expiratory pressure (PEEP) was set according to predefined criteria [1–3]. Mean tidal volume (± standard deviation) was 6.8 ± 0.9 ml/kg ideal body weight (469 ± 64 ml), respiratory rate was 29.5 ± 3.7 breaths/min, and the fraction of inspired oxygen was 82 ± 12%. After measuring respiratory mechanics, arterial blood gases, and hemodynamics, we decreased PEEP by 25–30% (other mechanical ventilation variables remained stable). We re-evaluated measurements (1 h later) focusing on the effects of PEEP reduction on respiratory mechanics, hemodynamics, and fluid balance in a 12-h window before and after the PEEP change.

Mean PEEP reduction by 29% significantly increased respiratory system compliance and reduced hypercapnia, while oxygenation (PaO_2_/FiO_2_) did not worsen (Table 1). PEEP reduction was not accompanied by lung de-recruitment, as oxygenation was not deteriorated. Rather PEEP reduction decreased lung overdistension as interpreted by the increase in respiratory system compliance and decrease in dead space ventilation (reduced PaCO_2_). Concerning hemodynamics, PEEP reduction was followed by a substantial decrease in noradrenaline dose, most likely indicating improvement in cardiac output that allowed decrease in vasopressor dose, while blood pressure remained constant.

**Table 1:** Respiratory and hemodynamic data in 17 mechanically ventilated patients with COVID-19, before and after PEEP reduction

	Before	After	Difference between before and after (95% CI)^**a**^	***P*** value
PEEP, cm H_2_O	15.6 ± 1.7	11.1 ± 1.9	4.5 (4.0 to 5.0)	< 0.001
PaO_2_/FiO_2_, mm Hg	115.8 ± 30.5	121.9 ± 26.3	− 6.1 (− 13.8 to 1.6)	0.11
PaCO_2_, mm Hg	49.7 ± 10.4	42.9 ± 5.6	6.8 (3.4 to 10.1)	0.001
Cst, ml/cm H_2_O	60.0 ± 8.3	63.2 ± 7.4	− 3.2 (− 4.9 to − 1.5)	0.001
Noradrenaline, μg/kg/min	0.21 ± 0.12	0.08 ± 0.08	0.13 (0.09 to 0.16)	< 0.001
MAP, mm Hg	67.2 ± 6.1	69.1 ± 2.6	− 1.9 (− 4.4 to 0.5)	0.11
Fluid balance, ml	1980 ± 764^b^	942 ± 331^c^	1039 (760 to 1317)	< 0.001

Data are expressed as mean ± standard deviation

^a^Data are expressed as mean difference [95% confidence interval (CI)]; ^b^fluid balance during 12 h before PEEP reduction; ^c^fluid balance during 12 h following PEEP reduction

*PEEP* positive end-expiratory pressure, *PaO*_*2*_ partial pressure of arterial oxygen, *FiO*_*2*_ fraction of inspired oxygen, *PaCO*_*2*_ partial pressure of arterial carbon dioxide, *Cst* static compliance of the respiratory system, *MAP* mean arterial pressure

Application of unnecessary high PEEP (i.e., when PEEP does not result in lung recruitment) increases transpulmonary pressures, forcing West Zone 3 lung regions to Zones 2 and 1. This phenomenon increases dead space ventilation, resulting in hypercapnia when minute ventilation remains constant. Pulmonary vascular resistance increases, leading to a variable degree of right ventricular dysfunction due to ventriculo-arterial uncoupling. PEEP effects may be exacerbated when the pulmonary vascular bed is injured, as in ARDS, where intravascular obstruction is observed due to thromboemboli [4]. A hypercoagulable state and extensive lung capillary thrombosis has also been reported in COVID-19 [5].

When lung compliance is relatively normal, substantial amount of the alveolar pressure is transmitted to the pleura. Therefore, relatively high PEEP in a non-recruitable lung with almost normal compliance may significantly increase pleural pressure and have a detrimental impact on hemodynamics by deteriorating venous return. A conservative or de-resuscitative fluid strategy is recommended in the management of patients with ARDS [6]. Yet, in our patients, a strategy of increased PEEP approximating the ARDSnet protocol was accompanied by substantial vasopressor dosage and 12-h fluid balance. PEEP de-escalation led to significant reduction of cumulative fluid balance during the following 12 h and a three-fold decrease of vasopressor dosage. Decreased need for vasopressors and better fluid management translates in increased cardiac output and organ perfusion accompanied with less fluid accumulation in the lungs.

COVID-19 lung involvement is unique having a “pneumonia pattern” than being a typical “ARDS pattern” at least in the initial phase during the first days after intubation, and this was rather the case in our patient group [5]. Therefore, mechanical ventilation should be applied differently from the routinely followed ARDS protocol. Intensivists have to be cautious in discriminating between pneumonia and typical ARDS. Failure to discriminate between COVID-19 pneumonia and ARDS and adoption of erroneous ventilatory strategy may have detrimental effects on hemodynamics, resulting in organ hypoperfusion and ultimately multiorgan failure.

## Data Availability

Drs. Ilias Siempos, Eleni Magira, and Professor Spyros Zakynthinos had full access to all of the data in the study. After publication, the data will be made available to others on reasonable requests after approval from the corresponding author (VT, vasotsolaki@yahoo.com) and Evangelismos Hospital, Athens, and University Hospital of Larissa.
